# Food-Grade Encapsulation Systems for (−)-Epigallocatechin Gallate

**DOI:** 10.3390/molecules23020445

**Published:** 2018-02-17

**Authors:** Meng Shi, Yun-Long Shi, Xu-Min Li, Rui Yang, Zhuo-Yu Cai, Qing-Sheng Li, Shi-Cheng Ma, Jian-Hui Ye, Jian-Liang Lu, Yue-Rong Liang, Xin-Qiang Zheng

**Affiliations:** 1 Tea Research Institute, Zhejiang University, Hangzhou 310058, China; 11616052@zju.edu.cn (M.S.); 11516051@zju.edu.cn (Y.-L.S.); 21616096@zju.edu.cn (X.-M.L.); 21616106@zju.edu.cn (R.Y.); 21716160@zju.edu.cn (Z.-Y.C.); qsli@zju.edu.cn (Q.-S.L.); jianhuiye@zju.edu.cn (J.-H.Y.); jllu@zju.edu.cn (J.-L.L.); 2 Liupao Tea Academy, Wuzhou 543003, China; zjumasc@aliyun.com

**Keywords:** food, proteins, carbohydrates, lipids, EGCG, in vitro digestion

## Abstract

(−)-Epigallocatechin gallate (EGCG) has attracted significant research interest due to its health-promoting effects such as antioxidation, anti-inflammation and anti-cancer activities. However, its instability and poor bioavailability have largely limited its efficacy and application. Food-grade materials such as proteins, carbohydrates and lipids show biodegradability, biocompatibility and biofunctionality properties. Food-grade encapsulation systems are usually used to improve the bioavailability of EGCG. In the present paper, we provide an overview of materials and techniques used in encapsulating EGCG, in which the adsorption mechanisms of food-grade systems during in vitro digestion are reviewed. Moreover, the potential challenges and future work using food-grade encapsulates for delivering EGCG are also discussed.

## 1. Introduction

Tea (*Camellia sinensis*) is the most widely consumed beverage in the world after water. A variety of bioactive ingredients including catechins in tea possess numerous health benefits such as antioxidant, anticancer, antidiabetic, antihyperglycemic, and UV-shielding effects [1,2,3,4,5,6,7]. Tea catechins are a group of bioactive polyphenols present in tea leaves, mainly comprising eight kinds of catechins, among which EGCG is the most abundant and shows the strongest bioactivity, making it an excellent candidate for application in drugs and functional food development [8]. However, the bioavailability of EGCG is relatively poor because of its instability and low permeability under the neutral or alkaline conditions of the gastrointestinal (GI) tract and also its active efflux effect [9,10]. Therefore, improving the digestive adsorption of EGCG and inhibiting its active efflux are considered to be an appropriate way to increase the bioavailability of EGCG to allow its nutritional functions to be enjoyed. 

In the medical and functional food field, numerous encapsulation systems are available for the controlled delivery of bioactives to improve their bioavailability [11]. Food grade materials are generally abundant resources with advantages of biodegradability, biocompatibility, functionality and low-cost, which show great potential for protectively delivering bioactive compounds through human GI tract to target tissues [12,13]. There is considerable interest in improving the flavor and increasing the bioavailability of EGCG using food-grade encapsulation systems. The present review aims at combining the encapsulation technologies and delivery efficiencies insights to highlight the fabrication methods and essential parameters that should be taken into consideration when developing encapsulates for protecting EGCG. Furthermore, some challenges and future research directions are also discussed in this review.

## 2. EGCG in Tea

**Figure 1:**
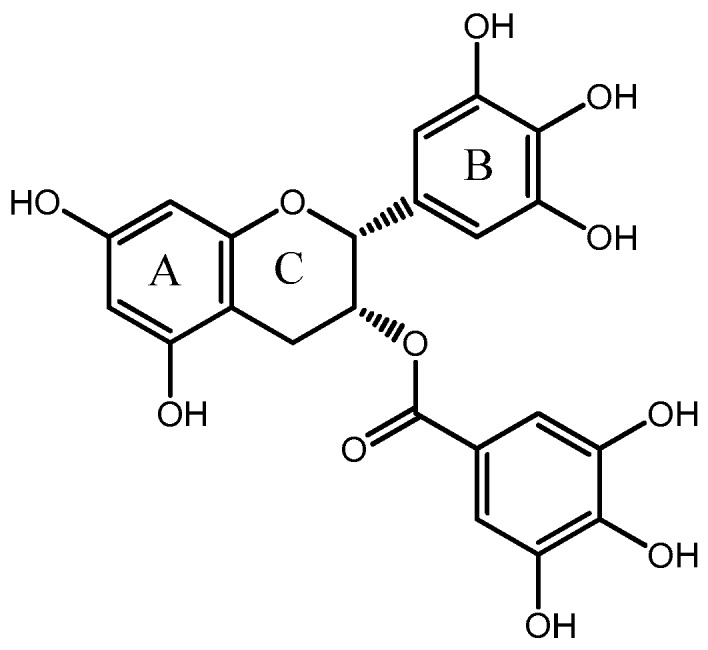
The chemical structure of EGCG.

EGCG is a key family member of catechins originating from tea. The content of EGCG is the highest among the catechins in green tea leaves. Structurally, EGCG is comprised of two aromatic rings conjugated by a three-carbon bridge, namely a C6-C3-C6 structure, with hydroxyl groups at carbons 3’, 4’, 5’ of the B ring and a gallate moiety esterifying carbon three of the C ring [14,15] (Figure 1). EGCG is rich in phenolic hydroxyl groups, which lead it possess health-beneficial activities. A number of in vivo and in vitro studies have been performed to show that EGCG has beneficial anti-oxidative, anti-inflammatory and anticarcinogenic properties [16,17,18,19,20,21]. 

However, the production or preservation stability of EGCG is poor, which is one of the primary limitations of EGCG applications. There are many factors influencing the stability of EGCG, such as light, temperature, pH, oxygen, EGCG concentration, and also the level of oxidants [22,23,24,25,26]. Auto-oxidation and epimerization are two significant reactions causing the instability of EGCG and leading to the partial loss of its health-promoting effects [27]. EGCG is a pH and temperature sensitive compound and at low concentration (such as 20–100 μM), it is apt to be oxidized under conditions of lower temperature (<44 °C), neutral and alkaline pH conditions. Meanwhile, when EGCG is at a high concentration (mM level or higher) and under higher temperature (>44 °C) and acidic (pH 2–5.5) conditions, it is apt to be epimerized to its isomer gallocatechin gallate (GCG) [9,27,28,29]. EGCG (300 mg/L) remains stable in solution at pH of 3–6 and 25 °C for 24 h, but 25% and 83% of it is lost if kept in pH 7 citrate buffer solution for 24 h at 4 °C and 25 °C, respectively [25]. When EGCG (500 μM) was kept at 60 °C and 90 °C for 8 h, respectively, 3.2% and 27.4% of the EGCG was epimerized into GCG [26]. The EGCG epimerization product GCG possesses similar health functions to EGCG, with no toxic effects, however, the oxidation of EGCG is usually accompanied with brown coloration and creaming, in which higher molecular weight compounds are formed through polymerization [26,27]. 

Moreover, when consumed EGCG is exposed to oral administration systems with acidity ranging from pH 1.5 in the stomach up to pH 8.5 in the intestine, where the main adsorption occurs [9]. It has been reported that the alkaline environment of intestinal digestion is one of the main reasons leading to its poor bioavailability [30,31,32,33]. In an in vitro simulated GF digestion model, EGCG recovery was between 6.1% to 12.1% under various tested conditions [31,34,35]. However, a high stability of EGCG was observed by adding ascorbic acid, with 29.6% loss [36]. EGCG is hydrophilic, and no specific receptors have been found on the surface of small intestinal epithelial cells to carry EGCG into cells. Generally, an apparent permeability coefficient (P_app_) > 1 × 10^−6^ cm/s means high permeation, while P_app_ < 1 × 10^−7^ stands for low permeation. The P_app_ for EGCG is as low as 0.8 × 10^−7^ to 3.5 × 10^−7^ cm/s [37,38,39]. EGCG is transported across the epithelium through passive diffusion, including paracellular and transcellular diffusion [40]. After absorption, EGCG can be extruded back into the lumen by active efflux. This efflux process is associated with efflux transporters including ATP-binding cassette transporters families such as p-glycoprotein (P-gps), multidrug resistance-associated proteins (MRPs), and breast cancer resistance protein (BCRP), which are distributed in the surface of the intestinal epithelial cell [41]. Studies have suggested that EGCG and its metabolites are pumped out by MRPs [37,40,42]. The efflux effects were inhibited in the presence of selective MRP inhibitors (indomethacin, probenecid, MK571), resulting in increasing P_app_ values, while the cellular uptake of EGCG in the presence of Pgp inhibitors (cyclosporine A, vinblastine, GF120918, indomethacin, probenecid) was not changed [37,40,42]. These results suggest that EGCG and its methylated metabolites are substrates of MRP1 and MRP2, but not a substrate of Pgp. It was this sensitivity and poor stability, passive diffusion and active efflux of EGCG in the GI tract that cause its low adsorption and bioavailability. Therefore, it is of great importance to develop new materials as edible EGCG biological carriers to improve its stability and bioavailability.

## 3. Food-Grade Encapsulation System

### 3.1. Proteins for EGCG Encapsulation

Food-grade proteins have been commonly used as hydrophilic emulsifiers to form oil-in-water emulsions. They are natural nutritional materials and show excellent aqueous solubility, emulsifying and film formation properties. Proteins, such as sodium caseinate, gelatin, whey protein, and zein proteins, are commonly used as wall materials for many polyphenols like EGCG. All proteins are amphiphilic and show different spatial structures and physical and chemical properties under various external conditions. The functional properties of the proteins may be modified or improved through appropriate processes [43], which extend their application for encapsulation. Crosslinking between proteins and EGCG could enhance the encapsulation. In recent years, proteins and peptides with superior properties were broadly used as encapsulation shell materials in the food industry. An extensive list of food-grade proteins used for encapsulating EGCG is given in Table 1.

Protein encapsulation usually occurs starting from emulsions. Milk proteins are effective encapsulating materials. Casein and whey are the major milk proteins. Milk proteins are frequently used in combination with polysaccharide and lipids. Sodium caseinate (0.5%) and soybean oil (10%) has been used in the formulation of nano-sized (230–250 nm) oil-in-water emulsions with EGCG at concentrations up to 6 mg/mL [44]. Sodium caseinate adsorbed at the oil-water interface can associate to high concentrations of EGCG. More than 90% of the EGCG was loaded at the interface when EGCG concentration was up to 2 mg/mL, and about 70% EGCG was adsorbed when the EGCG concentration was up to 5 mg/mL [44]. However, acidic conditions destabilized the emulsions, while addition of high methoxyl pectin (HMP) could improve its stability at low pH [45]. Sodium caseinate-stabilized emulsions can be employed as a platform for EGCG delivery, during which an emulsion of 240 nm size was obtained with a formulation of 0.35% sodium caseinate, 20% soybean oil, 0.45% HMP at EGCG levels between 0–6 mg/mL [46]. An in vitro digestion showed that the presence of EGCG reduced the extent of free fatty acid release during the duodenal phase, but sodium caseinate was fully hydrolyzed [46].

Whey protein isolate and bacterial cellulose were used to encapsulate hydrophilic or lipophilized EGCG using emulsion electrospraying with encapsulation efficiencies (EEs) ranging from 56% to 97% and particle sizes from 253 to 3226 nm [47]. The stability of EGCG in the particles during storage was influenced by the relative humidity (RH), pH and temperature. Low RH (26–53%) and neutral or alkaline pH (6–9) conditions were beneficial to protecting EGCG in the particles [47]. The non-capsulated free EGCG was completely degraded after storage at 37 °C for 7 days or at 60 °C for 4 days. The emulsion was completely degraded after storage at 37 °C for 25 days or at 60 °C for 20 days [47]. β-Lactoglobulin (β-LG) is the most abundant whey protein and wildly used as a natural delivery protein for bioactive components. β-LG (0.4%), l-carrageenan (0.6%) and canola oil (5%) stabilized oil/water (O/W) emulsion (~400 nm) was more stable with lower EGCG concentration (<0.5%) and the emulsion droplet size showed negligible changes within 14 days [48]. β-LG combined with chitosan can form smaller nanoparticles of 193.8 nm size to carry EGCG [49]. Recently, β-LG-chlorogenic acid complex showed better antioxidant activities than using β-LG alone, and EGCG loaded β-LG-chlorogenic acid nanoparticles (105–110 nm) better EEs ranging from 71.8 to 73.5% [50].

Besides milk proteins, other edible proteins are also used as wall materials for encapsulating EGCG. Gelatin shows remarkable gelation properties with different gel strength and chain length and is usually used to encapsulate gelatin/EGCG particles by a layer-by-layer assembly technique [51,52]. Food-grade gelatin EECG encapsulates were produced from the appropriate ingredients by electrospraying in dilute acetic acid, with high EGCG EE (96%) while avoiding the use of high temperatures and toxic solvents [53]. Hydrogels prepared from gelatin and γ-polyglutamic acid (γ-PGA) through ionic interaction presented sustained release of EGCG [54]. A comparative study on EGCG micro-hydrogels prepared using the biopolymers gelatin and chitosan showed that gelatin was more adequate as a wall material for the encapsulation of EGCG than chitosan, achieving higher EE (95 ± 6%), being more effective in delaying EGCG release and degradation in aqueous solution and exhibiting 7-times higher bioaccessibility of the EGCG than chitosan after in vitro gastrointestinal digestion. The low bioaccessibility of EGCG in chitosan was due to the neutralization of the carbohydrate in the basic simulated salivary conditions, which precluded subsequent flavonoid release. Also, the gelatin micro-hydrogels hindered dimer formation during in vitro digestion, resulting in increased bioavailability [55]. 

Zein, as a water-insoluble plant protein from a renewable natural source, has been used to form EGCG colloidal particles with sodium caseinate as a stabilizer in sizes ranging from 170 nm to 250 nm by the anti-solvent precipitation technique [56]. Zein/chitosan nanoparticle fabrication *via* evaporation and freeze drying showed a controlled-release of EGCG and protection of EGCG from oxidation [57]. The fabrication of ferritin-EGCG co-assemblies induced by urea (20 mM) enhanced the EGCG stability, with homogeneous state spheres being 12 nm in size and 17.6% in EE, in which the urea played an essential role in promoting the permeation of EGCG into the ferritin cage [58].

The techniques for encapsulating EGCG using food-grade proteins include emulsification, ionic gelation, freeze drying, electrospraying, self-assembly, layer-by-layer assembly, vacuum evaporation, precipitation and covalent grafting. The protein-encapsulated EGCG showed remarkably enhanced stability and bioavailability, and they can be considered multi-functional EGCG delivery systems.

### 3.2. Carbohydrates for EGCG Microencapsulation

Carbohydrates, including chitosan, cellulose polymers, starch-based materials, gum arabic and sodium alginate, are preferred as encapsulating matrices for many applications due to their good biocompatibility and biodegradability as well as non-toxicity. Their thermal and emulsification functional properties, are complex and it is necessary to choose the polymers properly and modifications such as starch modification are usually employed. Chitosan in particular is the most common encapsulation material for EGCG. Gum arabic and sodium alginate are by far the best wall materials for encapsulating hydrocolloids. Carbohydrates can stabilize formulations and provide barrier protection and controlled release of the bioactive compounds. Studies on encapsulating EGCG using carbohydrates are listed in Table 2.

When EGCG was incorporated into a gum arabic-maltodextrin matrix at a particle size 120 nm by homogenization and spray drying methods, the EE reached 85% and the antioxidant properties of EGCG were preserved [59]. By changing the proportion of food grade matrix and EGCG, the EE was increased as high as 96%, with a zeta-potential of −36 [60]. Homogenization is a simple, efficient and environmentally friendly method that can produce small and stable particles. EGCG-loaded nanoparticles fabricated by alginate and chitosan by high-pressure homogenization resulted in higher DPPH radical scavenging activity [61]. EGCG on particles prepared by gas saturated solution drying (PGSS-drying) using octenylsuccinate starch (OSA-starch), soybean lecithin and β-glucan, three distinct natural origin carriers, showed enhanced storage capacity and antioxidant activity, among which β-glucan and soybean lecithin systems were confirmed to improve the intracellular activity of EGCG [62].

Chitosan-based particles for encapsulating EGCG are commonly fabricated by two basic methods, i.e., self-assembly and ionic gelation. The self-assembly technique refers to the autonomous interaction between encapsulating agent and bioactive. EGCG-loaded nanoparticles prepared from chitosan and polyaspartic acid with a size of 102.4 nm effectively improved EGCG stability in body fluids and showed greater activity against rabbit atherosclerosis than free EGCG alone [63]. An encapsulation system using dextran sulfate-coated amphiphilic chitosan derivative-based nanoliposomes with a 64.5–189.8 nm size range via a layer-by-layer self-assembly technique exhibited excellent sustained release properties and protected EGCG from degradation during exposure to simulated intestinal fluid [64]. The EGCG nanoparticles composed of caseinophosphopeptide (CPP) and chitosan based on self-assembly and ionic gelation methods were mentioned in Table 2. Ionic gelation is also a widely used technique because it can be performed under mild conditions avoiding EGCG degradation. Among these studies, EGCG-loaded bioactive peptides chitosan particles prepared by ionic gelation presented smaller size (~150 nm) and relatively lower EE (70.5–81.7%) compared with those made by the self-assembly technique, whose size is around 300 nm, with EEs ranging from 84% to 90% [65,66,67]. Gallic acid grafted chitosan conjugates were found to show increased solubility in neutral and alkaline environments [65]. An in vitro digestion experiment showed EGCG controlled release or enhancement of intestinal permeability after encapsulation [65,66,67]. EGCG encapsulated in chitosan-tripolyphosphate nanoparticles with particle sizes of 440 nm enhanced the concentration of EGCG in the stomach and jejunum, resulting in an increased plasma exposure of EGCG [68]. 

Carboxymethyl chitosan (CMC) and chitosan hydrochloride (CSH) are different kinds of water-soluble chitosan with special structures containing carboxymethyl and amino groups. CMC and CSH form EGCG particles with larger sizes via ionic gelation [69,70]. Interestingly, EGCG loaded in CSH sulfobutylether-β-cyclodextrin sodium (CSH-SBE-β-CD) system displayed decreased antioxidant activity, which is considered to be related to the restriction of EGCG release and diffusion owing to the disappearance of the swollen rubbery matrix [69,71]. When chitosan was grafted with caffeic acid and ferulic acid and then assembled with CPP, the system showed high EE of EGCG and preventive effects against EGCG degradation under neutral or alkaline conditions [72].

### 3.3. Lipids for EGCG Microencapsulation

Lipids are often used as encapsulation material because of their crystallization, melt property and moisture barrier properties. There are three categories of lipids commonly used for encapsulation, i.e., hydrocarbon-rich substances, simple lipids, and lipid-derived substances. Lipids are often soluble in non-polar solvents and insoluble in water. However, liposomes can be made of a single type of phospholipids or by mixtures of different phospholipids, and are able to dissolve water-soluble and lipid-soluble molecules at the same time. For EGCG encapsulation, the lipid materials, encapsulation technique, and outcomes are shown in Table 3.

Liposome encapsulates of EGCG made using egg phosphatidylcholine (PC) and cholesterol via the organic solvent evaporation method, with average diameters of 378.2 nm and EE of 99%, effectively protected EGCG against degradation during GI tract transit [73]. EGCG nanoliposome (EN) particles with 180 nm size and EE of 85.8% were obtained by the reverse-phase evaporation method using PC and cholesterol [74]. The EN was more stable during in vitro digestion and effectively enhanced the inhibitory effect on tumor cell viability at higher concentrations [74]. The EN prepared by the ethanol injection combined with dynamic high-pressure microfluidization, with an average size of 71.7 nm exhibited a high EE 92.1%, and presented sustained release of EGCG. However, the zeta-potential was as low as −10.81, showing the EN was unstable [75].

EGCG in particles fabricated with cholesterol via ethanol injection exhibited stronger antioxidant ability than free EGCG [76]. EGCG-loaded niosomes had smaller particle size (~60 nm) than EGCG EN, but their EE (76%) was lower than that of EGCG EN [76]. EGCG microparticles formulated using soybean lecithin via a gas-saturated solution drying method improved the antioxidant activity and intracellular activity of EGCG [62]. EGCG in soy lecithin liposomes had high recovery rate after in vitro digestion [77,78]. EGCG liposomes fabricated using soy lecithin, glycerol monostearate and stearic acid by an emulsion-solvent evaporation method, with sizes ranging from 112.5 to 157.4 nm and EEs ranging from 67.2% to 89.5%, improved the stability of the interface and prevented agglomeration [79]. EGCG liposomes subjected to further incorporation of the EGCG liposomes by proliposome method into alginate and chitosan microparticles protected EGCG against degradation at alkaline pH values [80].

Solid lipid nanoparticles (SLN) and nano-structured lipid carriers (NLC) prepared via a homogenization and ultrasonic assistance technique were used as biocompatible nanocarriers for delivering EGCG [81,82]. Because SLN had a solid-state core, it differed from NLC. Both SLN and NLC had large size particles (300–369 nm), high stability (from 8 weeks to 3 months) and great potential for the controlled release of EGCG in the GI tract [81,82]. An EGCG oral delivery system based on NLC functionalized with folic acid could stimulate the expression of folate receptors, resulting in improvement of the intestinal permeability of EGCG [81].

Above all, liposomes are considered to have a prosperous future for EGCG encapsulation, and SLN and NLC are looked at as innovative edible delivery systems for EGCG. The methods used to form liposomes include homogenization, ultrasonic and freeze drying. To improve the properties of EGCG liposomes, non-phospholipids, including cholesterol and fatty acids, were usually added during liposome preparation. The barriers to be overcome in this field include the chemical instability and degradation involving hydrolysis, oxidation, aggregation and fusion.

### 3.4. Food Grade Systems for EGCG Delivery and Their Possible Mechanisms of Action

Food-grade encapsulation systems are considered to be an innovative strategy to improve the bioavailability of EGCG for oral administration. Simulated in vitro digestion models are usually used to evaluate the bioavailability of EGCG. In vitro modeling systems of oral phase, gastric phase, small intestine and colon can be simulated. EGCG released from encapsulated materials under GI conditions generally occurs through diffusion and destruction of the particles by digestion. Digestive enzymes play a vital role in EGCG oral delivery because EGCG could interact with salivary proteins and inhibit lipase and protease activity [83,84]. EGCG release in in vitro digestion systems after encapsulation is presented in Table 4.

The protective effects of EGCG encapsulated by food grade materials were systematically investigated. Sodium caseinate emulsions loaded with EGCG were subjected to three stages of in vitro digestion, in which extensive physical changes were induced by saliva components and fatty acid release was reduced in the duodenal stage [46]. β-LG-EGCG complex showed relatively faster EGCG release rates compared with β-LG complex with chitosan or chlorogenic acid. Chitosan/β-LG double walled structures improved the controlled release of EGCG because β-LG was resistant to proteolysis of pepsin and could be easily hydrolyzed by trypsin in neutral aqueous solution [49]. After the degradation of β-LG, the exposed chitosan can adhere to the intestinal wall to increase the residence time of EGCG in the intestinal tract, resulting in improved bioavailability [49]. β-LG-CA conjugate showed slow release rate and extent of EGCG through inhibiting the activity of digestive enzymes [50]. Delivering EGCG by zein and sodium caseinate tunes the release of EGCG by forming a coating layer during digestion, and encapsulating EGCG to zein-based system modulates the rate of fat digestion via Pickering emulsion stabilization effect and interaction between EGCG and lipase enzyme [56]. EGCG encapsulated by proteins inhibited the activity of lipase, and it has been suggested that EGCG reduce adipose tissue mass via lipase inhibitory effects [85].

Chitosan is the only alkaline natural polysaccharide, which possesses positive charge and therefore binds strongly to negatively charged surfaces. Chitosan has many biological functions and it was widely used in preparing EGCG encapsulates with unique properties, such as controlled release, mucoadhesion, permeation enhancing, and efflux pump inhibitory effects. It is documented that chitosan can penetrate across the small intestinal epithelium in two ways, i.e., transcellular and paracellular pathways [86]. Encapsulation of EGCG with dextran sulfate-coated amphiphilic chitosan derivative-based nanoliposomes showed a stabilizing effect on the EGCG. The residual EGCG was 69.6% and 51.1% after 1.5 h and 3 h, respectively, which indicated a significantly reduced degradation of EGCG in simulated intestinal fluid, showing stability-enhancing ability [64]. It was also reported that chitosan can enhance the plasma exposure of EGCG through an enhancement in concentrations of EGCG in the stomach and jejunum of mice [87]. Nanoparticles fabricated by chitosan and CPP enhanced the intestinal permeation of EGCG through Caco-2 cells model with a maximum P_app_ value 1.3 × 10^−6^ cm/s, which was significantly higher than the maximum P_app_ by non-encapsulated EGCG (3.5 × 10^−7^ cm/s) [67]. The increased penetration may be contributed by the opening of the cellular tight junction and the enhancement of intracellular transportation. It is shown that chitosan carriers can inhibit intestinal P-gp and improve the adsorption of bioactives [88]. The possible interactions between polymeric efflux pump inhibitors (like chitosan) and efflux pumps as follows: (a) inhibition mediated by ATP depletion, (b) inhibition mediated by interaction with the membrane, (c) bypassing bioactive efflux by a drug-polymer conjugate, (d) inhibition mediated by interfering with ATP-binding sites and, (e) blocking of drug binding sites or other sites within the trans-membrane domains [89]. EGCG-loaded chitosan-CPP nanoparticles could enter Caco-2 cells in dose and time dependent manner, and the intestinal permeability and absorption of EGCG were significantly enhanced as delivered by chitosan [90].

**Figure 2:**
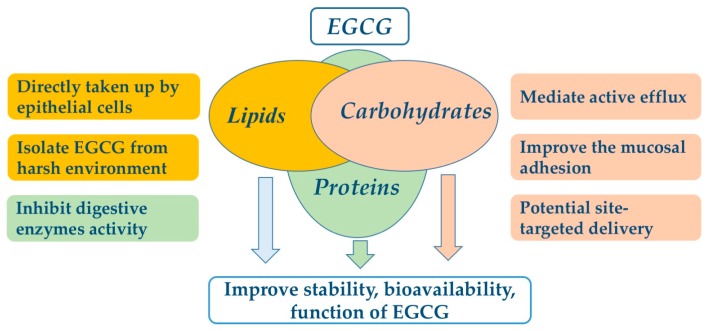
The potential mechanisms of food-grade encapsulate for improving EGCG bioavailability.

EGCG nanoliposomes and niosomes were used to overcome the chemical instability of EGCG and enhance its bioavailability. EGCG encapsulated in lipids was isolated from the external membrane environment by a lipid bilayer to decrease the environmental effects, such as oxygen level. In simulated intestinal fluid (SIF), the stability of EGCG was significantly improved by nanoliposome encapsulation. After 2 h of incubation, the residual EGCG encapsulated in nanoliposomes was 22.5% while non-encapsulated EGCG was not detected [75]. To improve the delivery efficiency of EGCG, nanoparticles can be coated with ligands such as folic acid to target the intestinal epithelial cells, with a 10% initial burst release in the first hours [81], and after 21 h, the maximum cumulative release of EGCG is 48% [81], suggesting the lipids are suitable encapsulation materials for EGCG, protecting it from degradation by harsh intestinal conditions and enhancing its bioavailability. EGCG-SLN enhanced the permeation and retention effect in cancer cells, which improved the anticancer activity compared to free EGCG [76]. Lipids encapsulated EGCG is gradually released under harsh conditions, which can be directly taken up by epithelial cells in the small intestine [91], resulting in improvement of EGCG bioavailability. The effects of encapsulation on improvement of EGCG stability and bioavailability are shown in Figure 2. 

## 4. Challenges

Despite marked advantages, inconsistency between the research work and industry application is observed in food grade delivery systems. There are many factors leading to this inconsistency. First, the number of studies on the loading efficiencies is limited. Only a few studies have presented the loading efficiencies, which range from 0.37% to 30% [50,52,62,68,80,82]. Second, it is difficult to compare the release of EGCG in different encapsulation systems owing to the differences in tested indicators of the different tests. There is no standard in vitro modeling method because the conditions involved in human digestion are complex and not completely elucidated yet [92]. There is a great difference in pH values in the reported in vitro intestinal digestion tests (Table 4). Third, there are many factors that influence the instability EGCG such as GI tract environmental conditions including pH, ion levels and enzymes, which affect the efficiency of EGCG delivery. The pH is a crucial factor, and it influences not only the stability of EGCG, but also the encapsulation. The particle size of sodium caseinate emulsions loaded with EGCG showed a decreased extent of flocculation at pH 5 compared with neutral pH [46]. During simulated intestinal digestion, the average diameter of EGCG-loaded niosomes increased from 60 nm to 178 nm in 2 h [76]. Nanoparticles formed by EGCG and chitosan showed a rapid increase in particle size from 103 to 856 nm when the pH value was increased from pH 3.5 to pH 7.4 [63]. Digestive enzyme, mucin and ions are also important factors inducing the aggregation during in vitro oral processing [46,49,76]. Finally, the effect of encapsulated EGCG should finally be confirmed by clinic studies. Oral administration of 400 mL of 1.25% green tea (134 mg of EGCG in total) by adults, the plasma EGCG concentration was about 80 nmol/L during a period up to 12 h after administration [93], which was much lower than the 50% inhibition concentration (IC_50_) of EGCG against α-amylase (about 52 μmol/L) [93] and against proliferation of HCT116 colon cancer cells (145 μmol/L) [94]. When male Swiss Outbred mice were orally administered EGCG encapsulated in chitosan nanoparticles (0.76 mg/kg, equating to 45.6 mg per 60 kg adult animal), the plasma EGCG concentration was 37.8 nmol/L after 1.5 h of administration [87], which was still far below the previous IC_50_ values.

## 5. Conclusions and Expectations

EGCG has been confirmed to have broad beneficial health activities in cell and animal models, indicating it is a promising functional foodstuff for supplementation. However, its sensitivity to diverse environments, poor stability in GI passive diffusion and active efflux are major factors leading to the low bioavailability of EGCG. The stability, bioavailability and function of EGCG can all be improved by encapsulation using food grade materials such as proteins, carbohydrates and lipids. EGCG can be encapsulated in proteins by emulsification, ionic gelation, freeze drying, electrospraying, self-assembly, layer-by-layer assembly, vacuum evaporation, precipitation and covalent grafting. EGCG encapsulated in proteins shows a sustained release due in part to the inhibition of the activity of digestive enzymes. EGCG can be encapsulated in carbohydrates by homogenization and spray drying, gas saturated solution dry, self-assembly and ionic gelation. The EGCG incorporated into carbohydrates showed improved mucoadhesion, intestinal permeation, site-targeting delivery and inhibition of active efflux. EGCG can be encapsulated in lipids as SLN or NLC via homogenization, organic solvent evaporation and dynamic high-pressure microfluidization. The EGCG encapsulated in lipids showed improved stability and sustained release, and it can be directly taken up by epithelial cells. The loading efficiency in the encapsulating systems remains to be improved, and future work should be focused on the improvement of loading efficiencies in various materials, and the development of new materials to be used to stabilize EGCG during storage and enhance EGCG site-controlled release. Moreover, clinical translation of the encapsulated EGCG should be investigated and the related mechanisms should be theoretically demonstrated. Overall, encapsulating EGCG with food grade materials could improve the bioavailability and functionality of EGCG, which have the potential to be wildly applied in functional foodstuff sector.

## Figures and Tables

**Table 1:** Overview of studies on the effect of protein based encapsulation on EGCG.

Wall Material	ET	APS (nm)	EE (%)	ZP	PI	Activity	Reference
Sodium caseinate	Emulsion	230~250	/	−41~−43	/	/	Sabouri et al., 2015 [44]
Sodium caseinate Pectin	Emulsion	240	/	/	/	No inhibition of proteolytic; Reduce free fatty acid release	Sabouri et al., 2017 [46]
Whey protein; Bacterial cellulose	Emulsion; electrospray	253~3226	56~97	/	0.5~2.9	Protection against moisture, heating and dissolution during storage	Paximada et al., 2017 [47]
β-LG; l-carrageenan	Emulsion	~400	/	/	/	Enhanced in vitro anticancer activity	Ru et al., 2010 [48]
β-LG; Chitosan	Ionic gelation	100~500	54.2~60.7	10~35	0.13	Controlled release of EGCG	Liang et al., 2016 [49]
β-LG-chlorogenic acid	Covalent grafting; freeze dry	105~110	71.8~73.5	−44~−48	/	Protection against degradation or oxidation	Fan et al., 2017 [50]
Gelatin	Self-assembly	50~300	/	/	/	Retain antioxidant activity; High loading (30%, *w*/*w*)	Shutava et al., 2009 [51,52]
Gelatin	Electrospray	470	96	/	/	Increase in vitro antioxidant activity	Gomez-Mascaraque et al., 2015 [53]
Gelatin; γ-PGA	ionic gelation	/	62.7~68.7	/	/	Sustained release of EGCG	Garcia et al., 2014 [54]
Gelatin	Electrospray	~5000	95	/	/	Delay EGCG release and degradation; Protection against oxidation	Gomez-Mascaraque et al., 2016 [55]
Zein; Sodium caseinate	Antisolvent; precipitation	170~250	37~46	−30~−42	<0.15	Control EGCG release and fat digestion	Donsi et al., 2017 [56]
Zein; Chitosan	Vacuum evaporation; freeze dry	156~241	61.4~80.7	21.1~35.1	0.09~0.23	Protection against oxidation	Liang et al., 2017 [57]
Ferritin; Urea	Self-assembly	12	17.6	/	/	Improve EGCG stability	Yang et al., 2017 [58]

ET: encapsulation technique, EE: encapsulation efficiency, APS: average particle size, ZP: zeta potential; PI: polydispersity index; γ–PGA: γ-polyglutamic acid; /: no data.

**Table 2:** Overview of studies on the effect of carbohydrates based encapsulation on EGCG.

Wall Material	ET	APS (nm)	EE (%)	ZP	PI	Activity	Reference
Gum arabic-maltodextrin	Homogenization; spray dry	40~400	96	−36	0.58	Preserve EGCG antioxidant properties	Peres et al., 2011 [59]
Gum arabic-maltodextrin	Homogenization; spray dry	120	85	−12.3	0.45	Retain EGCG inhibitory effects on prostate cancer cells proliferative	Rocha et al., 2011 [60]
Alginate; chitosan	Homogenization	293	80.1	+37.49	/	Higher DPPH radical scavenge activity	Park et al., 2016 [61]
OSA-starch	Precipitation; GSSD	2000	80.5	/	/	Higher storage ability; Higher antioxidant activity	Goncalves et al., 2016 [62]
β-Glucan	Precipitation; GSSD	20,900	77.4	/	/	Higher storage and antioxidant ability; Improve EGCG intracellular activity	Goncalves et al., 2016 [62]
Chitosan; polyaspartic acid	Self-assembly	102.4	25	+33.3	0.224	Improve the ability against rabbit atherosclerosis	Hong et al., 2014 [63]
Amphiphilic chitosan; dextran sulfate; cholesterol	Self-assembly	64.5~189.8	90.8~95.1	+40.6~+51.8	0.323~0.422	Sustaining release and protect EGCG from degradation	Zou et al., 2015 [64]
CPP; chitosan; gallic acid	Self-assembly; freeze dry	~300	84~90	/	/	Controlled release; Prevent degradation and amplify anticancer against caco-2 cells	Hu et al., 2015 [65]
Bioactive peptides; CPP; chitosan	Ionic gelation	143.7	70.5~81.7	30.8	0.08~ 0.13	Controlled release and increase cellular antioxidant	Hu et al., 2013 [66]
Chitosan; CPP	Ionic gelation	150	/	32.2	0.05~0.14	Enhance EGCG intestinal permeability	Hu et al., 2012 [67]
Chitosan; tripolyphosphate	Ionic gelation	440	/	25	/	Enhance EGCG concentration in stomach, jejunum and plasma of mice	Dube et al., 2010 [68]
CSH-SBE-β-CD	Ionic gelation	150~12,000	/	−5~+30	/	Decrease antioxidant activity	Liu et al., 2016 [69]
CMC; folate	Ionic gelation	401.3	75	+36.6	/	Greater tumor inhibition effect	Liang et al., 2014 [70]
Caffeic acid; chitosan; CPP	Ionic gelation	273.8	88.1	+27.9	0.268	Controlled release; Prevent EGCG degradation under neutral or alkaline	Hu et al., 2016 [71]
Ferulic acid; chitosan; CPP	Ionic gelation	251.3	90.4	+25.7	0.386	Controlled release; Prevent EGCG degradation under neutral or alkaline	Hu et al., 2016 [71]

ET: encapsulation technique, EE: encapsulation efficiency, APS: average particle size, ZP: zeta potential; PI: polydispersity index; GSSD: gas saturated solution drying; CSH: chitosan hydrochloride; SBE-β-CD: sulfobutylether-β-cyclodextrin sodium; CPP:caseinophosphopeptide; CMC:carboxymethyl chitosan.

**Table 3:** Overview of studies on the effect of lipids based encapsulation on EGCG.

Wall Material	ET	APS (nm)	EE (%)	ZP	PI	Activity	Reference
Egg; PC, cholesterol	Organic solvent evaporation	104.6~378.2	84.6~99	−0.9~−26.2	/	Protection of EGCG against degradation; Increase EGCG uptake by tumor	Fang et al., 2006 [73]
PC; cholesterol	Reverse-phase evaporation	180	85.79	/	/	More stable *in vitro* digestion; Modulate the growth of Caco-2 tumor cells	Luo et al., 2014 [74]
Phospholipid, cholesterol	Ethanol injection; DHPM	71.7	92.1	−10.8	0.286	Sustained release of EGCG	Zou et al., 2014 [75]
Cholesterol	Ethanol injection	~60	76.4	/	0.110	Increase in vitro digestion antioxidant ability	Liang et al., 2016 [76]
Soybean lecithin	Precipitation gas saturated solution drying	8100	75.8	/	/	Higher storage ability and higher antioxidant activity; Improve the intracellular activity of EGCG	Goncalves et al., 2016 [59]
Soy lecithin	Homogenization	153~173	53.1~70.9	−42.4~−46.1	/	High retention in a low-fat hard cheese system; High recovery from digestion	Rashidinejad et al., 2014, 2016 [77,78]
Soy lecithin glycerol monostearate; stearic acid	Emulsion-solvent evaporation	112.5~157.4	67.2~89.5	−30.1~−37.2	0.14~0.268	Higher cytotoxicity against human breast cancer and prostate cancer cells	Radhakrishnan et al., 2016 [79]
Phospholipon 90 G, alginate, chitosan	Proliposome method; freeze dry	/	97.5	/	/	Higher stability in alkaline medium	Istenic et al., 2016 [80]
NLC; Folate	Homogenization; ultrasonic freeze dry	359	85	−28	0.18	Controlled release of EGC; Storage stability up to 8 weeks	Granja et al., 2017 [81]
SLN	Homogenization; ultrasonic	364	83	−24	0.19	Stable for at least 3 months; High stability and a slower release in vitro digestion system	Frias et al., 2016 [82]
NLC	Homogenization; ultrasonic	300	90	−28	0.15	Stable for at least 3 months; High stability and a slower release of EGCG in vitro digestion system	Frias et al., 2016 [82]

ET: encapsulation technique; EE: encapsulation efficiency; APS: average particle size; ZP: zeta potential; PI: polydispersity index; PC: phosphatidylcholine; NLC: nanostructured lipid carriers; SLN: solid lipid nanoparticle; DHPM: dynamic high-pressure microfluidization.

**Table 4:** EGCG release in vitro digestion system after encapsulation.

Encapsulation System	In Vitro Digestion System	EGCG Release in Vitro Digestion	Reference
β-LG; Chitosan	GP: pH 2.0 IP: pH 6.8	GP: % (t): ~30% (2 h) IP:% (t): ~70% (4 h)	Liang et al., 2016 [49]
β-LG-chlorogenic acid	GP: pH 1.5 IP: pH 6.5	GP: % (t): 14.2% (3 h) IP: % (t): 32.7% (4 h)	Fan et al., 2017 [50]
Gelatin	GP: pH 3 IP: pH 7	GP: RSA% (t): 23% (2 h) IP: RSA% (t): 36% (2 h)	Gomez-Mascaraqueet al., 2016. [55]
Chitosan	GP: pH 3 IP: pH 7	GP: RSA% (t): 15% (2 h) IP: RSA% (t): 5% (2 h)	Gomez-Mascaraque et al., 2016. [55]
Zein; sodium caseinate	GP: pH 1.2 IP: pH 7.4	GP: % (t): ~90% (2 h) IP: % (t): ~90% (2 h)	Donsi et al., 2017 [56]
Amphiphilic chitosan; dextran sulfate; cholesterol	IP: pH 7.4	IP: % (t): 63%(2 h) 51% (3 h)	Zou et al., 2015 [64]
Chitosan; CPP	GP: pH 1.2 IP: pH 7.4	GP: % (t): ~45% (2~8 h) IP: % (t): 35~40% (2 h) 32~25% (3~8 h)	Hu et al., 2015 [65]
Chitosan; CPP; gallic acid	GP: pH 1.2 IP: pH 7.4	GP: % (t): ~40% (2~8 h) IP: % (t): 35~40% (2 h) 32~25% (3~8 h)	Hu et al., 2015 [65]
Chitosan; CPP	GP: pH1.2	GP: % (t): ~35% (2 h) ~40% (4–8 h)	Hu et al., 2016 [72]
Chitosan; CPP; caffeic acid	GP: pH 1.2	GP: % (t): ~25% (2 h) ~30%(4–8 h)	Hu et al., 2016 [72]
Chitosan; CPP; ferulic acid	GP: pH 1.2	GP: % (t): ~30% (2 h) ~35% (4–8 h)	Hu et al., 2016 [72]
PC; cholesterol	GP: pH 1.3 IP: pH 7.5	GP: % (t): 21% (4 h) IP: % (t): 35% (4 h)	Luo et al., 2014 [74]
Phospholipid; cholesterol	GP: pH 1.2 IP: pH 7.4	GP: % (t): 94% (3 h) IP: % (t): 94~7.8% (0~3 h)	Zou et al., 2014 [75]
Cholesterol	GP: pH 2.0 IP: pH 7.4	GP: % (t): 99% (1 h) IP: % (t): 49% (2 h)	Liang et al., 2016 [76]
NLC; folic acid	GP: pH 1.6 IP: pH 6.5	GP: % (t): 13% (3 h) IP: % (t): 19~48% (3~21 h)	Granja et al., 2017 [81]

IC: Initial concentration of the encapsulated particle; RM: release media, % (t): % of EGCG released from total nominal EGCG in time t; RSA % (t): % of radical scavenging activity in time t; GP: gastric phase; IP: intestinal phase; CPP: caseinophosphopeptide; PC: phosphatidylcholine; NLC: nanostructured lipid carriers; /: no data.
